# Communication

**Published:** 1899-07

**Authors:** E. V. Wilcox

**Affiliations:** Office of Experiment Stations, Washington, D. C.


### COMMUNICATION.

In the *New York Medical Journal* for December 10, 1898, as a leading article (pp. 835–840), appeared an account by L. H. Warner, M.D., on the cultivation of *plasmodium malariæ*. On reading the article we were surprised at some statements concerning the development of mosquitoes. These statements are as follows:

"Dr. Walter F. Scheele, of New York City, recently conducted a number of experiments and investigations in mosquito development which proved that there are three distinct types of mosquitoes, each possessing a distinct degree of poisoning power in its sting. His claims are that mosquitoes originate and develop in foul water, especially when vegetable or animal albuminous substances are present. In the first stage of its development the mosquito is a conglomerate mass of different bacteria and microbes, formed by decomposing matter composed of vegetable and animal albumin which has been slowly abstracted from dead plants or animal substances. The latter, being in a state of decomposition, is a deadly poison. In the second stage of development the mosquito appears in wormlike shape with some power of motion. In the third stage it appears in ball-form, keeping constantly turning round. In the fourth stage it comes to the surface of the water fully developed excepting the wings, which soon grow; and at the last stage the most important point is to be noticed. Upon emerging from the water the mosquito is charged with a surplus of albuminous poison which must be got rid of immediately or death occurs; hence it instinctively seeks to preserve itself by stinging and injecting the injurious albumin into the only objects that will receive it, man and beasts."

At first we thought this curious travesty of science a huge joke, but the author seems to imagine that such stuff is real science. Are we to understand that the author saw Dr. Scheele's developmental stages of the mosquito? Here is a scientific romance which would discount the *Arabian Nights*. From decomposing animal substances bacteria and microbes are produced abiogenetically; from a combination of these microbes, we are told, a worm is produced "with some power of motion;" the worm is metamorphosed into a ball which is

"constantly turning round;" out of the ball is developed an adult mosquito. We were almost moved to tears by the pathetic tale about the condition of the mosquito upon leaving the water. The poor mosquitoes are said to be so full of poison that they have to inject it into man and beasts in order to save their own lives. Why could not the mosquitoes get rid of the poison by expectorating? When medical courses are made to include a little work on the fundamental principles of biology we hope there will be less paper spoiled by such dribble as this concerning the development of insects. It is difficult to understand how an editor of a reputable journal can allow such material to pass. If a mass of bacteria can develop into a worm, then into a ball, and finally into a mosquito, we certainly would not be surprised to hear of the plasmodium of malaria turning into a rattlesnake or a pine-tree. The author says Dr. Scheele ended his investigations with the remarkable discoveries which we have quoted. It is a pity that he did not complete the life-cycle of the mosquito. It would be interesting to know whether those New York mosquitoes lay eggs or bacteria, or both.

E. V. Wilcox,
Office of Experiment Stations, Washington, D. C.

